# Short Report: Race and Ethnicity Misclassification in Kidney Transplantation Research

**DOI:** 10.1097/TXD.0000000000001373

**Published:** 2022-09-16

**Authors:** Amber B. Kernodle, Valerie Thompson, Xiaomeng Chen, Silas P. Norman, Dorry L. Segev, Tanjala S. Purnell, Mara McAdams-DeMarco

**Affiliations:** 1 Department of Surgery, Johns Hopkins University School of Medicine, Baltimore, MD.; 2 Department of Medicine, University of Michigan, Ann Arbor, MI.; 3 Department of Surgery, NYU Grossman School of Medicine and NYU Langone Health, New York, NY.; 4 Department of Epidemiology, Johns Hopkins Bloomberg School of Public Health, Baltimore, MD.

## Abstract

Recently, the misuse of race as a biological variable, rather than a social construct, in biomedical research has received national attention for its contributions to medical bias. In national transplant registry data, bias may arise from measurement imprecision because of the collection of provider-perceived race rather than patients’ own self-report.

**Methods.:**

We linked Scientific Registry of Transplant Recipients data to a prospective, multicenter cohort study of adult kidney transplant patients (December 2008–February 2020) that collects patient-reported race. We computed Cohen’s kappa statistic to estimate agreement between provider-perceived and patient-reported race in the 2 data sources. We used an unadjusted generalized linear model to examine changes in agreement over time.

**Results.:**

Among 2942 kidney transplant patients, there was almost perfect agreement among Asian (kappa = 0.88, 95% confidence interval [CI], 0.84-0.92), Black (kappa = 0.97, 95% CI, 0.96-0.98), and White categories (kappa = 0.95, 95% CI, 0.93-0.96) and worse agreement among Hispanic/Latino (kappa = 0.66, 95% CI, 0.57-0.74) and Native Hawaiian/Other Pacific Islander categories (kappa = 0.40, 95% CI, 0.01-0.78). The percent agreement decreased over time (difference in percent agreement = –0.55, 95% CI, –0.75 to –0.34). However, there were differences in these trends by race: –0.07/y, 95% CI, –0.21 to 0.07 for Asian; –0.06/y, 95% CI, –0.28 to 0.16 for Black; –0.01/y, 95% CI, –0.21 to 0.19 for Hispanic/Latino; –0.43/y, 95% CI, –0.58 to –0.28 for White categories.

**Conclusions.:**

Race misclassification has likely led to increasingly biased research estimates over time, especially for Asian, Hispanic/Latino, and Native Hawaiian/Other Pacific Islander study populations. Improvements to race measurement include mandating patient-reported race, expanding race categories to better reflect contemporary US demographics, and allowing write-ins on data collection forms, as well as supplementing data with qualitative interviews or validated measures of cultural identity, ancestry, and discrimination.

## INTRODUCTION

Recently, the misuse of race as a biological variable, instead of a social construct, in biomedical research and in clinical decision-making tools used to estimate kidney function and inform clinical decisions related to timing of transplant referral, evaluation, wait listing, and approval of living kidney donors and allocation of kidneys has received national attention.^1^ Specifically, contemporary kidney function estimation tools founded on erroneous, historic beliefs that the dichotomization of Black versus non-Black in a correction factor contained meaningful biologic information has led to an underestimation of chronic kidney disease burden among Black patients and contributed to delayed referral and wait listing of Black patients with chronic kidney disease.^2-6^ Much of the discourse around race correction in kidney function estimation has focused on social construct definition and the consequences of imprecision.^7^ Less commonly addressed, however, is measurement imprecision of race.

Race is a multidimensional, relational social construct that is often collected by self-report or perceived by an observer.^8^ More specifically, provider-perceived race is a social assignment that is based on a variable combination of physical appearance (eg, skin color, hair texture, nose, and lip shape) and interpersonal interactions (eg, language, surname, attire) and varies according to observer demographics and familiarity with different racial groups.^9^ Perceived race is associated with differential exposure to racism, with Hispanic and American Indian individuals who are perceived to be White and lighter-skinned Black individuals reporting higher health status than those who are perceived to be their actual self-identified race or are darker-skinned.^8,10-13^ Self-reported race is how one identifies when presented with closed-ended survey instruments and is recommended in the Office of Management and Budget’s Standards for the Classification of Federal Data on Race and Ethnicity and used in vital statistics and population-based research.^14^ Although race is routinely present in kidney transplant (KT) registry data and commonly used in research, race measurement according to its unique dimensions has not been examined. It is possible that agreement between provider-perceived race and self-reported race may differ across commonly used data sources used to study KT patients.

Therefore, the goal of this study was to examine race measurement in kidney transplantation research. We used national, transplant registry data and data from a prospective, multicenter cohort of adult KT candidates and recipients (1) to quantify race agreement between provider-perceived race and patient self-reported race and (2) to examine the change in this agreement over time according to race classification.

## PATIENTS AND METHODS

### Study Population

This study leveraged 2 prospective cohorts of 2443 adult (18 y and older) patients with end-stage renal disease who were waitlisted for a KT and 1244 adult living and deceased donor KT recipients at Johns Hopkins Hospital, Baltimore, MD (candidates enrolled November 2009–February 2020, n = 2443; recipients enrolled December 2008–February 2020, n = 1244) and the University of Michigan University Hospital (recipients enrolled March 2015–November 2016, n = 83). After excluding duplicate patient records (ie, patients who were waitlisted and then transplanted), a total of 2942 patients with complete records were included in the analysis.

We linked each patient record to the Scientific Registry of Transplant Recipients (SRTR). The SRTR data system includes data on all donors, waitlisted candidates, and transplant recipients in the United States, submitted by the members of the Organ Procurement and Transplantation Network (OPTN). The Health Resources and Services Administration, US Department of Health and Human Services‚ provides oversight to the activities of the OPTN and SRTR contractors. This dataset has previously been described elsewhere.^15^

All research activities being reported are consistent with the Declaration of Helsinki and the Declaration of Istanbul. The study protocol was approved by the Johns Hopkins University Institutional Review Board and the University of Michigan Institutional Review Board. All cohort study participants provided written informed consent.

### Classification of Race and Ethnicity

At cohort enrollment, baseline characteristics and year were collected using a standard data collection form. The patient’s self-reported race was ascertained using a multiple-choice question with options that included White, Black, Asian/Asian American, Native Hawaiian/other Pacific Islander, and Other. An additional question was used to assess Hispanic ethnicity. For both race and ethnicity questions, patients were permitted to select 1 option only.

Transplant registry data on race is derived from OPTN data collection forms that are used by transplant hospitals, histocompatibility laboratories, and Organ Procurement Organizations. The OPTN collects provider-reported race and ethnicity in 1 question and permits the selection of >1 option. SRTR converts this data into race categories that include White, Black/African American, American Indian/Alaska Native, Asian, Native Hawaiian/other Pacific Islander, Arab/Middle Eastern, Indian Subcontinent, Hispanic/Latino, and Other and a separate ethnicity category that includes categories Hispanic/Latino and Non-Hispanic-Latino. In this study, we considered patients categorized as White, Black, Black/African American, American Indian/Alaska Native, Asian, Native Hawaiian/other Pacific Islander to be non-Hispanic. We reclassified patients who were American Indian/Alaska Native, Arab/Middle Eastern, or Indian Subcontinent in the SRTR database into other to make congruent with cohort data for analysis.

### Analysis

For analysis, the construct of race collected in the prospective cohorts was considered “patient self-reported race and ethnicity,” and that reported in the transplant registry was considered “provider-perceived race and ethnicity.” We computed the Cohen kappa statistic to estimate the agreement beyond expected by chance. A kappa coefficient of 0 indicates chance agreement, whereas 1 indicates perfect agreement, specifically ≤0 poor, 0.01 to 0.20 slight, 0.21 to 0.40 fair, 0.41 to 0.60 moderate, 0.61 to 0.80 substantial, and 0.81 to 0.99 almost perfect agreement.^16^ We then estimated the percent agreement between patient’s self-reported race and provider-perceived race for the overall study period using a pairwise Pearson correlation. After visually checking the distribution of observed agreement over time, we used unadjusted generalized linear models to assess the mean change in agreement per year.

All analyses were performed using Stata‚ version 15 (StataCorp, College Station, TX). Two-sided *P* values <0.05 were considered statistically significant.

## RESULTS

We identified 2942 patients, of which 81.1% (n = 2385) were waitlisted candidates and 18.9% (n = 557) were KT recipients. Of the total population, 4.1% (n = 120) were Asian, 42.3% (n = 1245) were Black, 3.4% (n = 101) were Hispanic/Latino, 0.2% (6) were Native Hawaiian or Other Pacific Islander, and 47.7% (n = 1404) were White individuals (Table S1, SDC, http://links.lww.com/TXD/A445). The overall agreement between patient self-reported race and ethnicity and transplant registry provider-perceived race and ethnicity was 95.3%. Percent agreement was 99.0% for Asian, 98.6% for Black, 98.1% for Hispanic, 99.8% for Native Hawaiian, 97.3% for White, and 97.8% for Other populations (Table 1).

**TABLE 1.** Agreement between patient self-reported race and ethnicity and provider-perceived race and ethnicity in kidney transplant patients (n = 2942) between 2009 and 2020

Race/ethnicity	Patient self-reported race prevalence (%)	Provider-perceived race prevalence (%)	Observed agreement	Kappa coefficient (95% CI)
Overall	–	–	95.3	0.92 (0.91-0.93)
Asian	4.1	4.8	99.0	0.88 (0.84-0.92)
Black	42.3	43.2	98.6	0.97 (0.96-0.98)
Hispanic	3.4	2.3	98.1	0.66 (0.57-0.74)
Native Hawaiian or other Pacific Islander	0.2	0.1	99.8	0.40 (0.01-0.78)
White	47.7	49.5	97.3	0.95 (0.93-0.96)
Other	2.2	0.1	97.8	0.08 (−0.01 to 0.17)

Self-reported race/ethnicity was collected in a prospective cohort study of kidney transplant candidates and recipients. Provider-perceived race/ethnicity was obtained from the Scientific Registry of Transplant Recipients. Cohen’s kappa coefficients with 95% CIs were presented.

CI, confidence interval.

For the overall study population, the kappa agreement was almost perfect (kappa = 0.92, 95% confidence interval [CI], 0.91-0.93). After stratifying by race category, we found that the kappa agreement was almost perfect for Asian (kappa = 0.88, 95% CI, 0.84-0.92), Black (kappa = 0.97, 95% CI, 0.96-0.98), and White (kappa = 0.95, 95% CI, 0.93-0.96) populations. The kappa agreement was substantial for the Hispanic population (kappa = 0.66, 95% CI, 0.57-0.74), fair for Native Hawaiian or Other Pacific Islander (kappa = 0.40, 95% CI, 0.01-0.78), and slight for Other (kappa = 0.08, 95% CI, –0.01 to 0.17) (Table 1).

**FIGURE 1.**
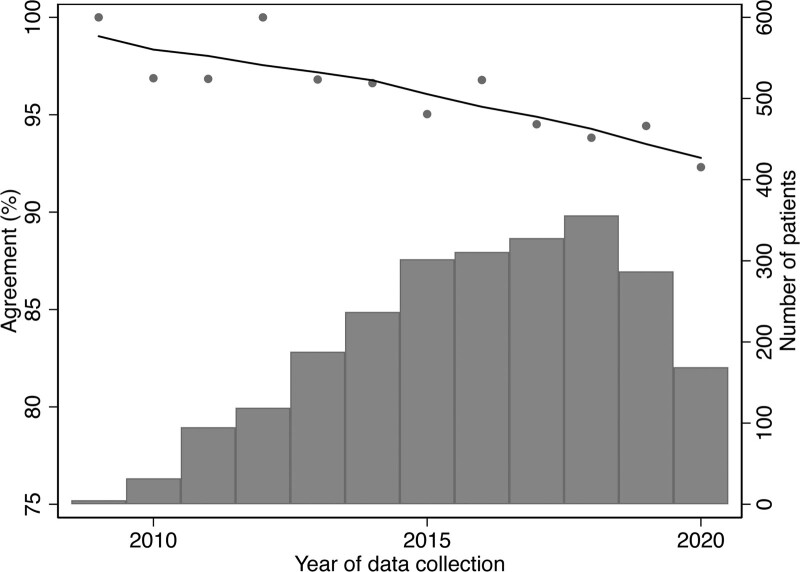
Observed agreement between patient self-reported race and ethnicity and provider-perceived race and ethnicity and number of patient records by year of candidate data collection (n = 2942) during 2009 to 2020. The observed agreements by year of candidate data collection in the national registry were depicted as the scatter points. The relationship between agreement and year was examined by Lowess plot.

**FIGURE 2.**
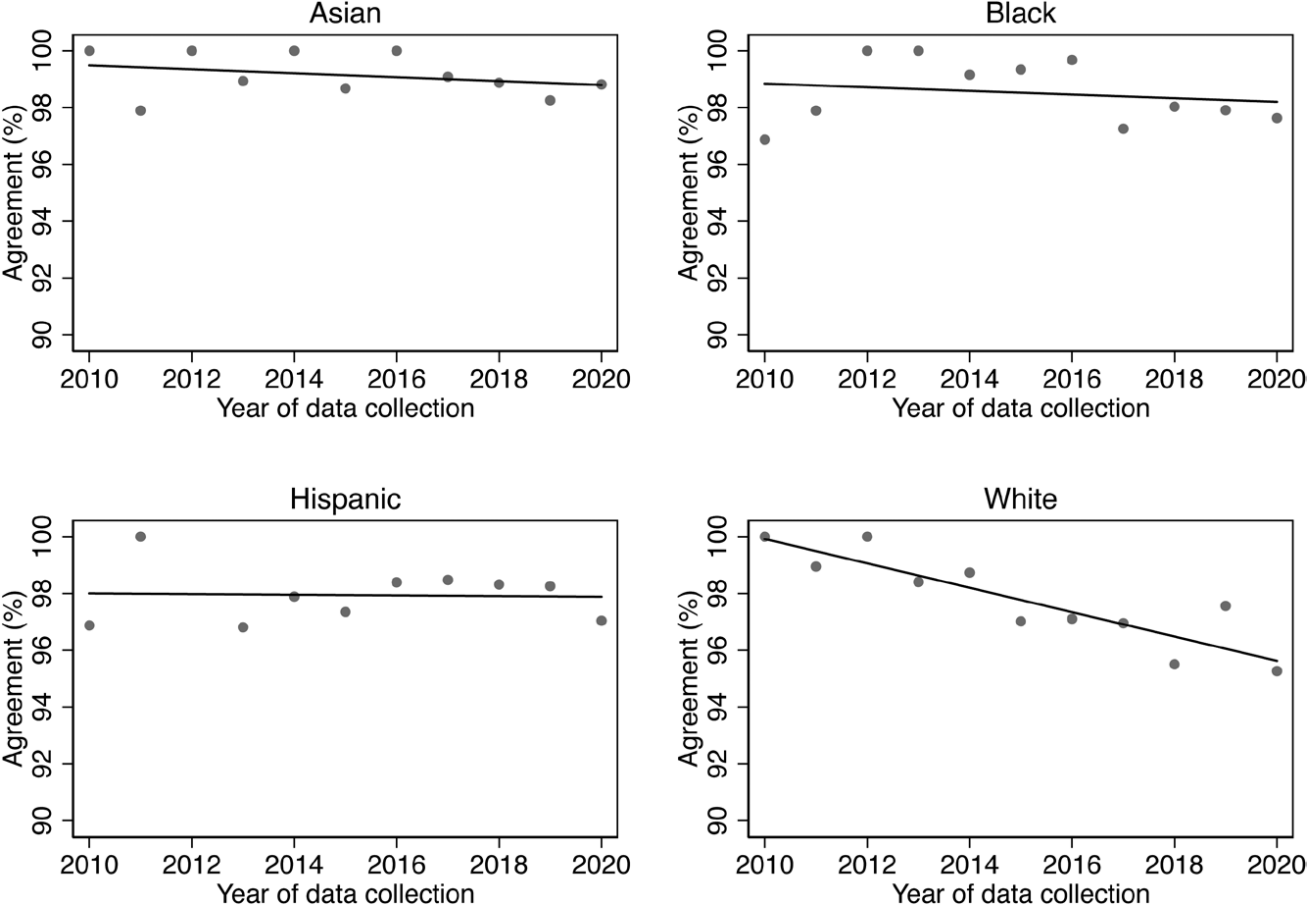
Observed agreements between patient self-reported race and ethnicity and provider-perceived race and ethnicity over time during 2009 to 2020. For each race/ethnicity, the observed agreements by year of candidate data collection in the national registry were depicted as the scatter points, and the trend over time was presented as a fitted line.

Agreement between patient self-reported race and provider-perceived race declined linearly over time (Figure 1), with a strong correlation between percent agreement and data collection year (r = –0.86, *P* < 0.001). Using an unadjusted linear regression model, we found that the percent observed agreement decreased 0.55% per year (difference in percent agreement = –0.55, 95% CI, –0.75 to –0.34). According to race, the changes in percent agreement per year were –0.07% per year for Asian (difference in percent agreement = –0.07, 95% CI, –0.21 to 0.07), –0.06% per year for Black (difference in percent agreement = –0.06, 95% CI, –0.28 to 0.16), –0.01% per year for Hispanic/Latino (difference in percent agreement = –0.01, 95% CI, –0.21 to 0.19), and –0.43% per year for White (difference in percent agreement = –0.43, 95% CI, –0.58 to –0.28) categories (Figure 2).

## DISCUSSION

In this study comparing prospective, multicenter cohort data to national transplant registry data, we found racial differences in agreement between patient self-reported and provider-perceived race, with almost perfect agreement being observed among Asian, Black, and White populations and worse agreement among Hispanic/Latino and Native Hawaiian/Other Pacific Islander populations. We also found that agreement between patient self-reported and provider-perceived race decreased linearly over time.

Our finding of relatively higher race misclassification among patients who are not classified as White when race from administrative data sources compared with self-reported race is consistent with prior studies.^17-22^ In a study comparing Medicare claims race data to self-reported race collected during a home healthcare visit, race was misclassified for 7.5% of the overall population and was greatest among Hispanic populations.^17^ Racial misclassification has also been found to vary across settings‚ with better agreement being observed in geographic regions with high concentrations of racial and ethnic minority individuals.^21^ In states with high concentrations of Hispanic populations, race variable imputation using surname improved race classification from 7.5% to 69.4% as compared with administrative data alone. Prior studies incorporating large, commonly used administrative data sources, such as Medicare claims and Surveillance, Epidemiology, and End Results program‚ have found misclassification to be associated with immigration status, preferred language, marital status with presumed surname changes, and educational attainment.^18,22^

We also found a novel finding that race misclassification has been worsening over time. Temporal trends in race misclassification could reflect the increasing number of multiracial US citizens and decreasing proportion who identify as White alone,^23^ changes in US political or social context over time affecting patients’ willingness to identify as part of certain racial groups, or outdated data collection procedures that inappropriately combine patients from unique social hierarchies. One of the most recently debated inaccuracies is the classification of Middle Eastern and North African (MENA) as White in Census data and other administrative data sources. In a study using an online, crowdsourcing platform to obtain a nonprobability of sample respondents and conduct a factorial experiment, Maghbouleh found that respondents who identify as MENA change their responses based on survey options, with 88% identifying as MENA alone when given the option.^24^

Our work is not without limitations. Although it is certain that race is self-reported in the prospective cohort, it is not certain whether transplant centers require patients to confirm their preferred race classification for inclusion in the national transplant registry, and this is an insurmountable limitation in this retrospective study. It is likely that race ascertainment in the registry is a combination of perceived and self-reported race. Given that this nondifferential measurement error biases toward the null, our findings are likely an overestimation‚ and racial misclassification is more problematic than reported. Both data sources used in this study collected race using closed-ended survey instruments, which can differ from open-ended, self-identified race. We treated self-reported race as the gold standard for data collection, but there is no gold standard race measurement. Both self-reported race and perceived race are valid social constructs for applicable research hypotheses.^8^ For example, perceived race is important for research questions concerning the influence of discrimination on health outcomes, whereas self-identified race is more important for understanding social networks, behaviors, and attitudes.^8^ We included multicenter, prospective cohort data that included an “Other” category in data collection, which is uninformative and inconsistent with contemporary guidance on the reporting of race and ethnicity in medical and science journals. The “Other” category in the cohort study likely captures American Indian or Alaska Native and/or multiracial populations, and prior research has demonstrated racial misclassification with geographic variation like what is observed among Hispanic/Latino populations.^20,21^ The absence of this population in our study, though unfortunate and contributing to historic marginalization, does not influence our findings and is a target for improvement and investigation in future research. Finally, it is possible that our finding of less-than-perfect concordance between the 2 data sources may also reflect data entry errors rather than different race measurement procedures. Notwithstanding, we are likely underestimating the magnitude of information bias present in kidney transplantation research.

Our work is important because race misclassification in national data in kidney transplantation has likely introduced information bias into surveillance reports and research evaluating access to and outcomes of transplantation. Prior studies have found that race misclassification in national, administrative data sources underestimates cancer cases by 16% to 37% for Asians^18^ and cancer incidence in Native Americans^20^ and distorts the magnitude of racial disparities in maternal and child health indicators used in national health statistic reports.^25^ Prior studies have also found qualitative differences in effect estimates after adjustment for misclassification.^26^ Although misclassification of race is not unexpected given its dynamic and inherently imperfect social construction that hinges upon person, place, and time, there are strategies that can be used to mitigate information bias. Moving forward, improvements in using registry-based race variable might include acknowledging the limitations of race definition and categorization in manuscripts, expanding race and ethnicity categories beyond federal administrative standards to reflect contemporary US demographics, including sensitivity analyses accounting for race misclassification,^27^ supplementing data with interviews to allow for patient self-report, including a write-in line on data collection forms, or including validated measures more specific to the research hypothesis of how race influences health outcomes such as skin tone, cultural identity, geographic ancestry, and everyday racism or discrimination experiences.^28,29^

In this study comparing race measurement in national, transplant registry data to a multicenter, prospective cohort collecting patient self-reported race, we found that race classification was different across the 2 data sources for the same patients. Misclassification of race has likely led to increasingly biased kidney transplantation research estimates over time, especially for studies including populations who are classified as Asian, Hispanic/Latino, or Native Hawaiian/Other Pacific Islander.

## ACKNOWLEDGMENTS

The data reported here have been supplied by the Hennepin Healthcare Research Institute as the contractor for the SRTR. The interpretation and reporting of these data are the responsibility of the author(s) and in no way should be seen as an official policy of or interpretation by the SRTR or the US Government.

## Supplementary Material


