# Supplementary material for: Short Report: Race and Ethnicity Misclassification in Kidney Transplantation Research
Source: Transplant Direct. 2022 Sep 16;8(10):e1373. doi: 10.1097/TXD.0000000000001373 (PMC9529064; doi:10.1097/TXD.0000000000001373)
Supplement: Supplementary file 1 [file txd-8-e1373-s001.pdf]

Supplemental Table 1: Cross-tabulation of patient self-reported race and ethnicity and provider-perceived race and ethnicity in kidney transplant patients (n=2,942) during 2009-2020. Self-reported race was collected in a prospective cohort study of kidney transplant candidates and recipients. Perceived race was obtained from the Scientific Registry of Transplant Recipients (SRTR).

**Self-reported race and ethnicity**	**Perceived race and ethnicity**						Total
	Asian	Black	Hispanic	Native Hawaiian or Other Pacific Islander	White	Other	
Asian	115 (3.9%)	1 (0.0%)	1 (0.0%)	0 (0.0%)	3 (0.1%)	0 (0.0%)	120 (4.1%)
Black	1 (0.0%)	1,238 (42.1%)	0 (0.0%)	0 (0.0%)	6 (0.2%)	0 (0.0%)	1,245 (42.3%)
Hispanic	3 (0.1%)	14 (0.5%)	56 (1.9%)	0 (0.0%)	28 (1.0%)	0 (0.0%)	101 (3.4%)
Native Hawaiian or Other Pacific Islander	4 (0.1%)	0 (0.0%)	0 (0.0%)	2 (0.1%)	0 (0.0%)	0 (0.0%)	6 (0.2%)
White	4 (0.1%)	6 (0.2%)	2 (0.1%)	1 (0.0%)	1,390 (47.2%)	1 (0.0%)	1,404 (47.7%)
Other	13 (0.4%)	12 (0.4%)	8 (0.3%)	1 (0.0%)	29 (1.0%)	3 (0.1%)	66 (2.2%)
Total	140 (4.8%)	1,271 (43.2%)	67 (2.3%)	4 (0.1%)	1,456 (49.5%)	4 (0.1%)	2,942 (100.0%)
